# Accurate discrimination of conserved coding and non-coding regions through multiple indicators of evolutionary dynamics

**DOI:** 10.1186/1471-2105-10-282

**Published:** 2009-09-08

**Authors:** Matteo Rè, Graziano Pesole, David S Horner

**Affiliations:** 1 Dipartimento di Scienze Biomolecolari e Biotecnologie, Università degli Studi di Milano, Via Celoria 26, 20133 Milano, Italia; 2 Istituto Tecnologie Biomediche, Consiglio Nazionale delle Ricerche, via Amendola 122/D, 70125 Bari, Italia; 3 Dipartimento di Biochimica e Biologia Molecolare "E. Quagliariello", Università degli Studi di Bari, Via Orabona 4, 70126 Bari, Italia; 4 Dipartimento di Scienze Dell'Informazione, Universita degli Studi di Milano, Via Comelico 39-41 20135 Milano, Italia

## Abstract

**Background:**

The conservation of sequences between related genomes has long been recognised as an indication of functional significance and recognition of sequence homology is one of the principal approaches used in the annotation of newly sequenced genomes. In the context of recent findings that the number non-coding transcripts in higher organisms is likely to be much higher than previously imagined, discrimination between conserved coding and non-coding sequences is a topic of considerable interest. Additionally, it should be considered desirable to discriminate between coding and non-coding conserved sequences without recourse to the use of sequence similarity searches of protein databases as such approaches exclude the identification of novel conserved proteins without characterized homologs and may be influenced by the presence in databases of sequences which are erroneously annotated as coding.

**Results:**

Here we present a machine learning-based approach for the discrimination of conserved coding sequences. Our method calculates various statistics related to the evolutionary dynamics of two aligned sequences. These features are considered by a Support Vector Machine which designates the alignment coding or non-coding with an associated probability score.

**Conclusion:**

We show that our approach is both sensitive and accurate with respect to comparable methods and illustrate several situations in which it may be applied, including the identification of conserved coding regions in genome sequences and the discrimination of coding from non-coding cDNA sequences.

## Background

A fundamental assumption of the "comparative genomics" approach is that clues to the functional roles of stretches of genomic DNA might be inferred from patterns of sequence conservation between related organisms. Until relatively recently it was assumed that longer stretches of conserved sequences would consistently overlap coding regions as, with the exception of relatively few non-protein-coding genes such as ribosomal RNAs and tRNAs, transcribed non-coding RNAs were thought to be relatively rare and cis-acting DNA regulatory elements were believed in general to be rather short. Recent findings have challenged these assumptions. Long, highly conserved apparently non-transcribed cis-acting elements have been identified [1] and estimates as to the proportion of transcripts that do not encode proteins continue to rise [2]. Furthermore, other lines of evidence suggest that several genes encoding "conserved hypothetical" proteins, for which little or no evidence of expression at the protein level exists, may not in fact constitute protein coding regions [3].

While it is probable that representatives of most gene families found in nature have been characterized (at least at the sequence level), lineage specific gene families, genes, and exons - which may be incorporated into messages by alternative splicing and which may not be recovered by ab-initio predictors as components of optimal gene models, are not uncommon (e.g.[4-6]). In this context, comparisons between relatively closely related genomes can permit identification of novel exons or coding genes that exhibit low levels of similarity to annotated proteins [7]. The extent and degree of conservation of non-coding transcripts is only now being widely studied and reliable "independent" in-silico support for the coding nature of gene predictions and transcripts is thus highly desirable. Accordingly, several comparative genomics approaches for the identification of coding regions through the differentiation of the evolutionary dynamics of coding and non-coding sequences have been proposed (e.g. [7-11]). Such methods do not rely on the annotation of homologous sequences or the conservation of specific functional signals.

Several of these methods are based on the expectation that the ratio of rate (or number) of (conceptually) synonymous substitutions to the rate or (number) of (conceptually) non-synonymous substitutions will be higher for genuinely coding sequences [10,11]. Mignone et al. [7] proposed a measure of coding potential derived from the product of the synonymous/non-synonymous substitution rate ratio and a measure of perceived similarity of peptides potentially encoded by the sequences under examination. A hybrid method which implicitly uses measures of amino acid similarity and synonymous/non-synonymous substitution rates as well as non-comparative information (such as dicodon usage frequencies) has been implemented in the software CRITICA [12] which, while designed for gene discovery in prokaryotes, can be adapted for the discrimination of coding and non-coding RNAs in eukaryotes [13].

Here we present a machine learning approach for the discrimination of coding and non-coding Conserved Sequence Tags (CSTs) generated through local alignment of genomic sequences or transcripts. Our method calculates various statistics related to the evolutionary dynamics of two aligned sequences - which need not correspond to entire genes or exons or models thereof. These features are considered by a Support Vector Machine which designates the alignment coding or non-coding with an associated probability score. Using a set of realistic genomic alignments that contains heterogeneous (partially coding) examples, we show that our method is both sensitive and accurate and illustrate several situations in which it may be applied, including the identification of conserved coding regions in syntenous genome sequences and the discrimination of coding from non-coding cDNA sequences.

## Results

### Data and annotation

To construct the training and validation sets, sequences from the ENCODE [14] regions of the human genome (excluding region Enm009 which contains many paralogous pseudogenes) were aligned with corresponding mouse syntenic regions as defined at ENSEMBL [15]. Both to avoid problems associated with erroneous insertions and deletions derived from sequencing errors, and to minimize heterogeneous alignment fragments (partially coding, partially non-coding) we analyse only the longest gap-free block in each HSP. We consider only gap-free blocks of length greater than 59 bases as shorter alignment fragments may be spurious (not reflecting homology relationships). Each gap-free alignment block (herein Conserved Sequence Tag or CST) recovered was annotated as coding if it was shown, through use of the Human RefSeq validated NP accessions) messages or Vega 46 (downloaded from ENSEMBL) annotated CDS, that at least 50% of the CST overlapped an annotated coding region. CSTs with between >0 and 50% overlap with coding regions and CSTs overlapping annotated pseudogenes were excluded from training and validation steps. Other CSTs were labelled non-coding.

The statistics we use in the discrimination between coding and non-coding conserved sequences are based on the evolutionary dynamics expected of coding regions, we therefore exclude gap-free alignment blocks with less than 5% variation between the aligned sequences as identical or nearly identical sequences suffer from the same stochastic limitations as very short alignment fragments.

Alignment of the ENCODE [14] regions with their syntenic counterparts in mouse yielded 7045 gap-free blocks conforming to the conditions specified above. However, of 3869 blocks that exhibited no overlap with annotated coding genes, 53 encoded putative peptides with significant similarity to UNIPROT entries. To minimize the use of potentially mis-annotated regions in the training and testing of the support vector machine, these blocks were excluded from subsequent experiments. The final dataset contained 6992 blocks. Preliminary studies using the feature values in isolation suggested that different features and different cutoff values for feature scores were discriminatory for different lengths of gap-free alignment blocks (see below) and that a the major part of this variation was associated with the behaviour of alignment blocks of length between 60 and 120 bases (not shown). Accordingly, all gap-free blocks considered were divided into short (60-119 bases) and long (120+ bases) categories.

3439 processed CSTs were between 60 and 119 nucleotides in length (2353 considered non-coding and 1086 with 50-100% overlap with annotated coding regions), while 3553 non-gapped regions of greater than 119 bases were recovered (of which 1463 were considered non-coding and 2090 exhibited 50-100% overlap with annotated coding regions). The datasets were divided randomly into training (60%) and validation (40%) sets.

### Analysis of discriminating power of features

We developed 17 measures of the coding potential of aligned homologous sequences (see Materials and Methods). Some of these measures derive purely from the characteristics of the individual sequences, while others are measures of the characteristics and patterns of variation in the alignment.

Support Vector Machine (SVM) is an effective classification method, but it does not directly estimate the feature importance.

The contribution of individual features to the discriminatory power of the trained SVM model can be assessed using an exhaustive search, by performing learning using each of the features separately, as well as all of the possible feature combinations, and then evaluating the performances of the trained classifiers. While this wrapper approach allows the possibility of accounting for feature interactions inside the trained model, it is also known to be prone to overfitting to the dataset used in the feature selection process. We thus chose to assess the predictor importance using a model free method. A ROC curve analysis was conducted for each predictor. A series of cutoffs was applied to the single features data to predict the coding/non-coding nature of the CSTs. Sensitivity and specificity were calculated for each cutoff value and the ROC curve was computed. Area under the curve (AUC) was then calculated for each predictor and used as measure of the feature importance.

Results of analyses performed independently for the long and short CST training sets are reported in Table 1A and 1B and show similarities in the ranking of the discriminatory capacity of the features tested between both datasets particularly for the most relevant predictors. However, among the less discriminatory predictors, notable differences in the rankings are observed between long and short datasets.

**Table 1:** Discriminating power of individual features

**Long CST training set**		**Short CST training set**	
**Feature**	**AUC**	**Feature**	**AUC**
CPS-ratio	0.9128	SpectAlign	0.8466
SpectrAlign	0.9113	CPS-best	0.8322
Ns/Nns-best	0.8538	CPS-ratio	0.8313
CPS-best	0.8532	Aasim-best	0.8259
GC-target	0.8263	Ns/Nns-best	0.8178
GC-probe	0.8231	GC-probe	0.7796
Ns/Nns-ratio	0.7948	GC-target	0.7727
AAsim-best	0.7893	AAID-best	0.7641
Stop-best	0.7854	Ns/Nns-ratio	0.7370
Codon-sim-ratio	0.7674	Aasim-ratio	0.6406
AAID-best	0.7222	AAID-ratio	0.6184
AAsim-ratio	0.6881	Codon-sim-ratio	0.6160
AAID-ratio	0.6626	GFB-ntID	0.6159
GFB-length	0.5587	GFB-length	0.6106
Tv/subs	0.5324	Stop-delta	0.6030
Stop-delta	0.5183	Stop-best	0.5915
GFB-ntID	0.5166	Tv/subs	0.5119

For each feature, Area Under Curve (AUC) calculated from ROC curves calculated with training sets was used as a measure of discriminating power of the feature (see results)

### Correlation between feature values

Many of the 17 features employed in the current study, either share elements of their mathematical formula (see Materials and Methods), or rely on characteristics of the genetic code such as partial redundancy concentrated at third codon positions. The degree of redundancy of information derived from pairs of features was evaluated using Pearson pairwise-correlations for both long and short training datasets.

**Figure 1:**
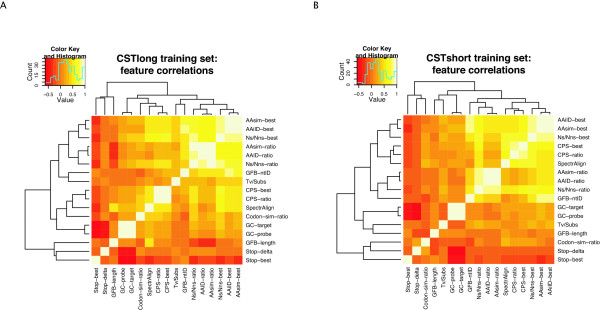
**Heatmap reporting the observed Pearson correlation coefficients between feature values for training sets**. Pearson correlation coefficients between feature values calculated on the long (A) and short (B) CST training sets are reported. The absolute counts of the number of pairwise correlation falling in any binned correlation range are provided in the heatmap key in histogram form.

Heatmaps shown in Figure 1A and 1B depict correlations between feature values for long and short datasets respectively, where features are also clustered according to correlation scores. The absolute frequencies of the number of comparisons falling into 15 binned correlation intervals are also shown as histograms in the heatmap keys (self correlations are reported in the frequency distributions). We note that the majority of pairwise comparisons between feature values yield low to moderate correlation scores - suggesting that information recovered by different features is not significantly redundant. Features describing stop codon frequencies (Stop-best and Stop-delta) show negative correlations with other feature values. This is because, for these features, low values are associated with high coding potential (while for the majority of features, higher values are expected to be associated with coding regions). While feature scores are not strongly correlated, Cochran-Mantel-Haenszel tests indicate strong interdependence of reading frames showing maximum scores, particularly for coding CSTs, indicating that features exploiting characteristics of the genetic code function in a consistent manner (see Additional file 1 and Additional file 1: Table S1).

### SVM training and validation

All feature values were scaled using the program svm-scale from the LIBSVM package [16]. Optimal values of the parameters C and G were estimated independently for long and short instances using the script grid.py provided with the LIBSVM distribution.

**Figure 2:**
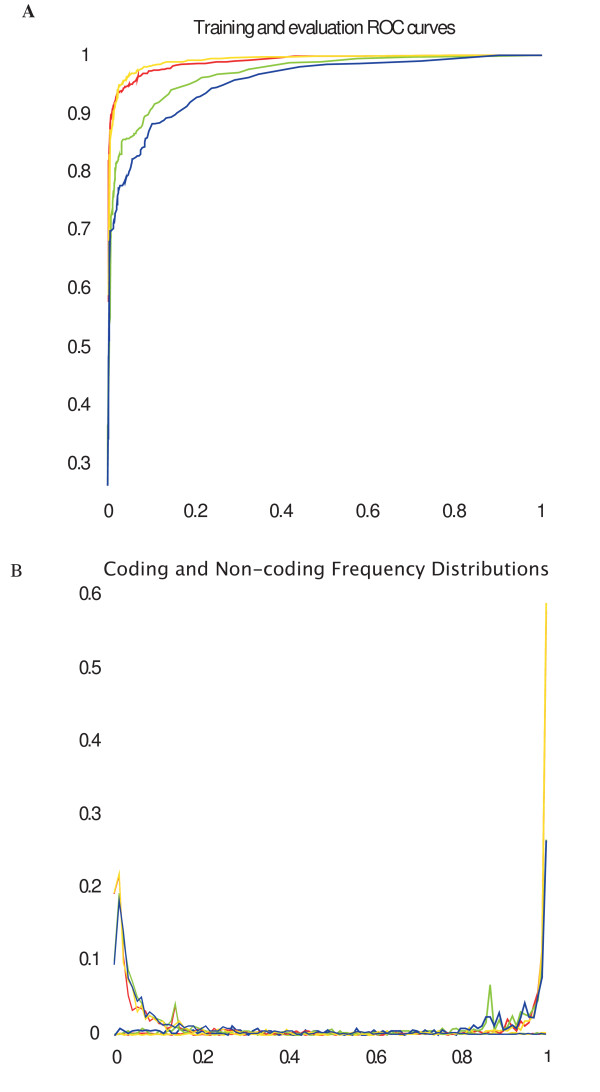
**SVM classification of Mouse/Human CSTS in training and validation**. A: ROC curves for long CST training (Yellow), long CST validation (Red), short CST training (Green) and short CST validation (Blue) experiments. B: P-score distributions of Coding and Non coding CSTs. The x axis represents the P-score generated by the SVM, the y axis shows relative frequency of CSTs by dataset (short coding, short non-coding, long coding and long non-coding) for training and validation phases of the experiment colour keys as in A, thick lines correspond to non-coding CSTs, fine lines correspond to coding CSTs.

The support vector machine was trained under conditions allowing the export of probabilities associated with predictions. ROC curves for training and validation of short and long CSTs are shown in Figure 2A where the large percentage of the area under the curves indicates the high overall accuracy of models generated, and the similarity of training and validation curves indicates the consistency of the models. Using the p-scores exported for each training instance cutoff values that allow recovery of 1% false positive results for both coding and non-coding categories were estimated on the training set (Table 2, Figure 2A) and allowed recovery of 86.5% of gap-free alignment blocks annotated as overlapping with coding regions (91.4% of "long" coding blocks and 74.0% of "short" coding blocks). 60.7% of gap-free alignment blocks annotated as not overlapping coding regions were recovered as non-coding (71.8% of "long" non-coding blocks, 53.7% of "short" non-coding blocks), while 24.6% of all gap-free blocks yielded indeterminate values under these post-processing conditions.

**Table 2:** Results of training and validation classification of alignment blocks at the 1% confidence interval

	**Training**	**Validation**
Long	2126	1427
Cod TP/total Cod	1139/1246 (91.42%)	768/844 (90.10%)
Cod FP/total NC	10/880 (1.14%)	8/583 (1.37%)
NC TP/total NC	632/880 (71.82%)	438/583 (76.13%)
NC FP/total Cod	13/1246 (1.04%)	5/844 (0.59%)
Indeterminate/total	332/2126 (15.62%)	208/1427 (14.58%)
		
Short	2059	1380
Cod TP/total Cod	481/650 (74.00%)	305/436 (70.00%)
Cod FP/total NC	14/1409 (0.99%)	7/944 (0.74%)
NC TP/total NC	757/1409 (53.72%)	498/944 (52.75%)
NC FP/total Cod	8/650 (1.23%)	8/436 (1.83%)
Indeterminate/total	799/2059 (38.81%)	562/1377 (40.72%)
		
Combined	4185	2807
Cod TP/total Cod	1639/1896 (86.54%)	1073/1280 (83.83%)
Cod FP/total NC	24/2289 (1.05%)	15/1527 (0.98%)
NC TP/total NC	1389/2289 (60.68%)	936/1527 (61.30%)
NC FP/total Cod	21/1896 (1.11%)	13/1280 (1.02%)
Indeterminate/total	1031/4185 (24.64%)	692/2807 (24.65%)

The model and post-processing parameters developed were used to analyse the validation set to demonstrate that model over-fitting was not evident (Table 2). 83.8% of gap-free blocks annotated as coding were recovered as coding (70.0% of "short" coding blocks and 90.1% of "long" coding blocks) with a combined false positive rate of 0.98%, while 61.3% of gap-free blocks annotated as non-coding were recovered as non-coding (76.1% of long non-coding blocks and 52.75% of short non-coding blocks) with a combined false positive rate of 1.02%. When false positive and false negative rates were both set at 1%, 24.7% of alignments yielded indeterminate scores.

### Analyses using SVM provides a significant improvement over discrimination with individual features

We calculated AUC scores for individual features and the trained SVM with the validation dataset (Additional file 1: Table S2). To determine whether the observed differences are significant we applied a non-parametric test based on the Mann-Whitney statistic [17] using the software StAR [18]. At the 1% confidence level, the SVM significantly outperformed all individual features for both the long and short validation datasets (see Additional file 1: Table S3). The results of all pairwise comparisons of discriminatory power of individual statistics are reported in Additional file 1: Table s4 and broadly confirm the patterns suggested by analyses presented in Tables 1A and B and Additional file 1: Table S2.

### Pseudogenes and paralogs

Our method uses only features expected to show biases in coding regions with respect to non-coding regions. As well as distribution of substitutions with respect to codon structure, we have shown that overall base composition and conceptual amino-acid similarity are strong indicators of the coding nature of aligned sequences. Accordingly, it is expected that alignments derived from pseudogenes will show characteristics of coding alignments. While ancestral pseudogenes might be expected to show less coding potential than cases where only one aligned sequence is pseudogenic, underlying compositional factors and residual conceptual amino acid similarity are expected to yield a number of false positive coding predictions. The evaluation of the impact of pseudogenes on the predictive power of our method is further complicated by concerns regarding the accuracy of pseudogene annotation. Non expressed pseudogenes my exhibit intact open reading frames, while the presence of premature stop codons is not guarantee that a sequence is not expressed at the protein level. Nevertheless, we have evaluated the coding potential of 290 alignments excluded from the training and evaluation sets because either human or mouse annotations implied an overlap with an annotated pseudogene. For long CSTs overlapping annotated pseudogenes, 88% (166 of 181) are evaluated as coding (90% of true coding regions in this size category were recognized as such) while 51% (51 of 99) of short CSTs that overlap annotated pseudogenes were classified as coding (vs 70% sensitivity for true short coding CSTs). These data are broadly consistent with our expectations: on the one hand they confirm, unsurprisingly, that pseudogenes retain certain characteristics of coding regions, on the other hand they indicate that our method can be used to assist in the identification and annotation of pseudogenes. The marked decrease in the recognition of short pseudogenic CSTs as coding with respect to the long category may also indicate that a proportion of the "pseudogenes" represented by long CSTs are in fact coding sequences - given the observed higher proportion of coding sequences among long alignments in general.

Local alignment of whole genomes (or syntenous regions) will result in the recovery of some HSPs derived from paralogous sequences. In general it is expected that the evolutionary dynamics of coding regions will be similar for paralagous and orthologous sequences. Our preliminary studies indicate that alignments of orthologous coding sequences tend to yield slightly higher coding probabilities than paralagous coding alignments, but that the marginal difference observed is likely to be due, mainly, to the fact that orthologous alignments tend to be longer than those derived from paralagous sequences (not shown).

### Quality of training data

**Figure 3:**
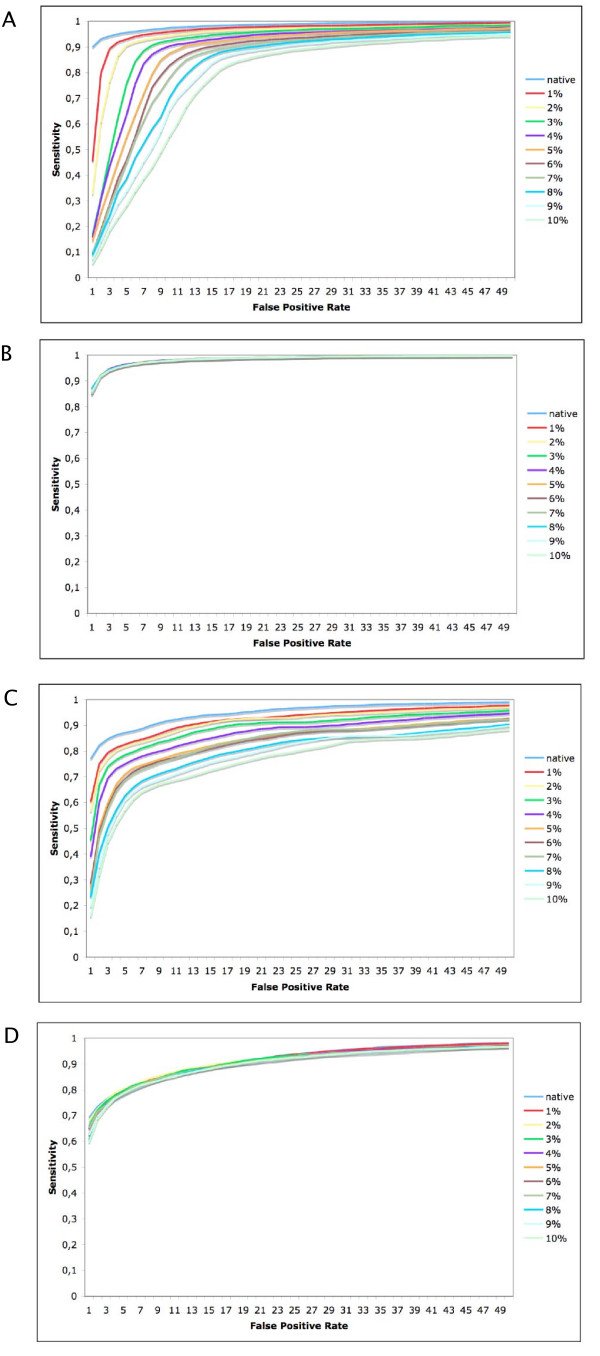
**ROC curves for training (A and C), and test (B and D) data, estimated when 0-10% of data annotations were randomly inverted in training**. For each proportion of inverted annotations, 10 jacknife replicates (70%) of training data were generated. SVM parameters were independently optimized for each jacknife set and values reported are means. The lack of change of ROC curves for test data indicate the stability of the SVM to errors in training annotations with the Human/Mouse data.

Errors in the annotation of training data are expected to negatively affect the performance of the method proposed here through several mechanisms. First, during the training of the SVM the hyperplane and margins generated by the SVM will be sub-optimal as the SVM attempts to generate a model which can classify the maximum possible number of points according to their a-priori annotation. The a-posteriori estimation of P-scores will also be influenced by mis-annotated instances. Finally, P-score cutoff values allowing one percent false positive predictions will be artificially high (or low for non-coding CSTs) as mis-annotated instances will tend to be recovered as false positive predictions. To assess the importance of the quality of annotation of the training data used, we performed experiments where a set proportion of the annotations (coding or non-coding) of the data points used in training and testing were deliberately but randomly inverted - in order to simulate the situation likely to be encountered in the development of the method with poorly annotated genomes. Jackknife experiments were performed with 70% of the training data where 1-10% of the annotations used were randomly inverted (10 replicates for each proportion of corrupted annotations). Data were scaled and SVM parameters were optimized independently for each replicate. ROC curves were plotted for the corrupted training data (Figure 3A and 3C). As expected, sensitivity at any given false positive rate - as well as the maximum sensitivity of the method - fall as the proportion of mis-annotated training instances rises. However, the fall in maximum sensitivity of the SVM is minimal (Figure 3A, C) and broadly corresponds with the expected proportion of coding CSTs mis-annotated as non-coding. With the native validation data, the ROC curves remain stable for both long and short CSTs (Figure 3B, D). We interpret these observations as suggesting that the SVM is relatively insensitive to the quality of training annotation and that the features used provide a strong separation between coding and non-coding CSTs.

### Quantity of training data

**Figure 4:**
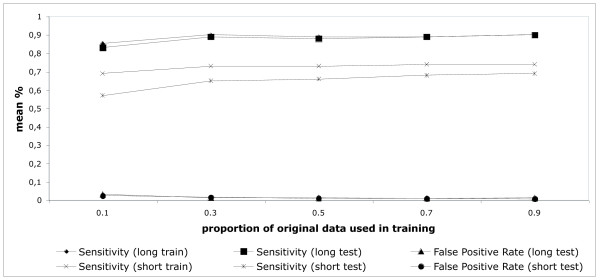
**Impact of size of training set on the classification of gap-free genomic alignment blocks**. For each proportion of the original training set used (x axis) 100 randomly selected training sets were generated and SVM parameters optimized independently. Sensitivity in training (1% false positive threshold) and sensitivity and false positive rates at the same threshold with validation data are shown. Blue = sensitivity long CSTs - training, Red = sensitivity long CSTs - test, Dark blue = False positive rate long CSTs - test, Yellow = sensitivity short CSTs- training, Green = sensitivity short CSTs - test, Orange = false positive rate short CSTs - test.

For each of a series of proportions of the original training dataset, subsamples were selected randomly 100 times and data scaling and parameter optimizations were performed for each subset of the original data. Cutoff values allowing 1% false positive coding predictions with the training data were established and each model generated was tested against the entire validation set. Figure 4 shows the average cutoff values, sensitivity with training and test data and false positive rates with validation data for each proportion of the training set tested. It is clear that with our data the training of the machine is relatively insensitive to the number of instances used - suggesting that, using the features developed here, a relatively small number of instances can adequately represent the diversity of evolutionary dynamics of coding and non-coding CSTs.

### Comparison of our approach with CSTminer

CSTminer [7,8] is an application previously developed by our group to identify CSTs and classify them as coding or non-coding based on their evolutionary dynamics. It relies on a single scoring function, very similar to the CPS score incorporated in our feature set (see Methods), that considers, for each conceptual reading frame, the synonymous and non-synonymous substitution rates and the perceived similarity of aligned amino acids encoded in each conceptual reading frame [7,8] threshold scores giving theoretical 1% false positive and false negative prediction rates were previously developed from distributions of coding potential scores derived from known coding and non-coding alignments. We have compared the performance of the new method with that of the algorithm used by CSTminer on our test set. The CSTminer algorithm showed an overall sensitivity of 71% with respect to coding CSTs (5% false positives) and 18% sensitivity with respect to non-coding csts (1% false positive), while 56% of all CSTs considered yielded indeterminate coding potential scores. ROC curves for CSTminer analyses of the test data are provided in Additional file 1: Figure S1. When compared to the performance of the current method with the same dataset (83.3% sensitivity for coding CSTs with 0.98% false positives, 61.3% sensitivity for non-coding CSTs with 1.02% false positives and only 24.65% of CSTs yielding indeterminate scores with these thresholds), the observed differences between the AUCs and non-parametric Mann-Whitney tests provided incontrovertible support for the hypothesis that the incorporation of multiple additional indicators of coding potential and the use of machine learning methods provides a significant advance in the discrimination of coding and non-coding alignments (Additional file 1: Figure S1). The marked decay of CSTminer performance with respect to values obtained in initial evaluations [7,8] is mostly due to the use of realistic alignments derived from genomic sequences. These alignments are often shorter than those used in previous CSTminer evaluations and are typically heterogenous with respect to their coding nature (partially coding).

### Discrimination of coding and non-coding RNA

To assess the utility of our method in the detection of coding transcripts we generated alignments with 1078 full-length human messages with corresponding sequences in Swiss-Prot used by Frith et al. [13] by Megablast against the murine REFSEQ mRNA collection. We were able to identify at least one gap-free block satisfying the conditions for analysis with our SVM (at least 60 bp, identity less than 95% identity) for 946 of the initial 1078 transcripts (5280 valid gap-free-blocks). Feature values were calculated for all these alignments in the same way as for genomic sequences and the feature values were scaled according to the genomic scaling ranges. After analysis with the SVM (using the genomic model parameters) longest gap-free blocks were classified as coding, non-coding or indeterminate on the basis of the probability cutoffs established in the previous sections. Of the 946 messages, 935 (98.8%) harboured at least one gap free alignment block that was designated as "coding", while 11 (1.16%) of the messages harboured only gap-free blocks classified as non-coding. We have compared our assessment of each human mRNA in the set studied, with the assessments generated for the same messages by Frith et al. [13] using 9 other methods .

Considering only messages for which usable alignments were generated (946 messages), our method is the most sensitive in the detection of their coding nature (Table 3). Additionally our method exhibits agreement with the majority assessment for a higher number of transcripts than any other method and for each transcript studied our method is, on average, in accord with 7.69 of the nine other methods (bettered only by BlastX with 7.71). Taken together, our data indicate that the approach presented here provides a good representation of the consensus of other methods designed to test the coding nature of transcripts, while escaping the explicit use of annotated homologs upon which BlastX is reliant. Frith et al. [13] conclude that consensus between methods provides a reliable assessment of the coding status of transcripts, and that a high proportion of messages reported as non-coding by a majority of methods, even among those with associated entries in SWISSPROT are unlikely encode real proteins. This last inference is supported by the generally poor level of experimental support for the expression of proteins encoded by transcripts evaluated as non-coding by the majority of methods [13] (a subset that matches extremely well with those recovered as non-coding by our method or for which we were unable to generate human/mouse alignments). In the current experiment we have restricted our method to the analysis of messages for which alignments with murine REFSEQ messages can be generated. Inclusion of other organisms would be expected to raise the sensitivity of our method further.

**Table 3:** Relative performance of methods to discriminate coding and non coding transcripts.

**Method**	**# coding**	**mean # methods in agreement (/9)**	**% agreement with majority**
This Work	935	7.69	98.83
longest ORF	788	7.05	83.56
BLASTX	923	7.71	97.88
rsCDS	924	7.64	97.77
Pfam	701	6.44	74.02
SUPERFAMILY	548	5.30	58.11
ESTscan	901	7.61	95.86
DIANA	907	7.54	95.97
CSTminer	724	6.55	76.78
CRITICA	898	7.57	95.55

946 human transcripts with associated Swissprot entries for which unambiguous homologs could be identified in the murine refseq collection were evaluated by the current method and 9 other approaches to discriminate coding from non-coding transcripts [13]. The table shows the number of transcripts evaluated as coding by each method, the mean number of other methods in agreement with the recovered evaluation and the percentage of transcripts for which each method agreed with the majority conclusion of the other methods

## Discussion

We present a new method for the discrimination of conserved coding sequences from conserved non-coding sequences exclusively on the basis of their evolutionary dynamics. This method is significantly more sensitive and selective than a previous approach developed in our group [7]. This is not surprising given that we use all of the information incorporated in the CSTminer algorithm (conceptual synonmymous to non synonymous substitution rate ratios and similarity of conceptually translated peptide products as well as a variety of other measures of evolutionary dynamics). Indeed, we show that the incorporation of multiple indicators of coding potential and the use of machine learning techniques results in a significantly more powerful approach than the use of individual metrics, including that used by CSTminer. We have used training and validation alignments that reflect the kind of data likely to be available to workers seeking to identify hitherto un-annotated coding regions to evaluate the proposed method. In particular, the data used in training and validation were generated from genome alignments and "coding" CSTs can be composed of coding and non-coding regions - avoiding over-estimation of the power of the method resulting from training and validation with entirely coding alignments. Several methods have been proposed for the estimation of the coding potential of transcripts [19,20]. These methods do not assess evolutionary dynamics of homologous sequences, but depend on measures of characteristics of potential open reading frames in transcripts, and, on the degree of similarity of putative translation products to annotated protein sequences. They thus risk failing to detect coding messages that represent members of genuinely novel gene families. Conversely, they are potentially prone to classifying transcripts as coding on the basis of similarity to transcripts incorrectly annotated as protein coding. Our method should thus be seen as complementary to these approaches in the discrimination of transcripts, and is more suited to the discrimination of predicted exons and alignments generated from genomic comparisons.

The method presented here is, used in isolation, not suitable for the fine-level definition of boundaries of coding and non-coding regions. However, we believe that information derived from this approach should be useful in directing experimental approaches for the confirmation of the coding status (and fine delineation of exon boundaries) of candidate novel coding regions.

While numbers of indeterminate conserved regions were comparable with other studies (around a quarter of all alignments), the method presented here consistently recovers around 85% of all coding CSTs (around 90% for blocks of length over 120 bases) with 1% false positive designations. Our approach is quick, produces a probability value indicating the confidence of the prediction and should be applicable to many different species pairs (providing that a sufficient number of well annotated pairs of conserved sequences between the pair of species is available for training). This approach might be expected to be particularly useful in the detection of coding regions whose homologs are poorly represented in nucleotide or protein databases (lineage-specific genes, genes/exons which are expressed only at low levels and genes coding regions which lack known protein domains).

## Conclusion

There is no reason to anticipate that the measures of evolutionary dynamics employed here should not be suitable for other species pairs and the pipeline proposed for the generation of training sets is easily transferable. We have also presented data suggesting that relatively few training instances are adequate for generation of a highly accurate discriminator and that the training of the SVM is relatively insensitive to the presence of mis-annotated CSTs. In the light of this observation, it is also tempting to generate more specialized classifiers, for example considering divisions of GC content of CSTs to account for known genomic context variations in synonymous substitution rates [21]. Other features apart from those proposed here are also under development and may easily be inserted into the method.

Here we have used 1% confidence limits to illustrate the sensitivity and specificity of the proposed methodology. While poor annotation of training instances carries the risk of distorting confidence limits estimated for our system, in the majority of experimental situations, attempts at validation will proceed from the highest confidence predictions and confidence intervals need not be employed.

## Methods

### Datasets/alignments/annotation

All alignments used in the current work were generated using discontiguous MegaBLAST with the options -e 1e-4 -D 1 -F "m D" -U T -J F -f T -t 18 -W 11 -A 5 0 -q -2 -G 5 -E 2.

The cDNA set consists of alignments (constructed with the aforementioned MEGABLAST parameters) of 1078 human messages encoding proteins listed in the SWISSPROT database [13] with the entire murine REFSEQ collection.

### Features

17 features related to the evolutionary dynamics of the aligned sequences were calculated for the longest gap-free block in each CST. These were:

1) Coding Potential Score (CPS): Similar to the Coding Potential score proposed by Mignone et al. [7]. For each possible reading frame in the longest gap-free block, we use the formula:



where Ns is the number of codons with synonymous substitutions, Nns is the number of codons containing non-synonymous substitutions, n is the total number of codons in the aligned block, and ΣAAsim is the total of similarity scores for aligned amino acids derived from a modified BLOSUM substitution matrix [7]. CPS-best is defined as the score associated with the highest scoring potential reading frame. CPS-ratio is defined as the highest scoring frame divided by the mean of the scores associated with the other 5 potential reading frames. We also consider individual values for ΣAAsim and Ns/Nns for each possible reading frame and record ΣAAsim-best (the highest ΣAAsim score), ΣAAsim-ratio (the highest ΣAAsim score over the mean of the scores associated with the other five possible reading frames), as well as Ns/Nns-best and Ns/Nns-ratio which are calculated as for ΣAAsim-best and ΣAAsim-ratio.

2) SpectrAlign: The discrete Fourier transform (DFT) of a genomic protein coding region of length N shows a peak at discrete frequency N/3. This feature, referred to as "3-periodicity", has been used in gene prediction algorithms [22,23]. If aligned sequences are protein-coding the spectral signal of the mismatches along the alignment is expected to be maximal at frequency N/3. For the alignment

query [i] =  [A T G A C T A A G A G A G A T C C G G]

      | | | | |   | |   | |   | |   | |

target [i] = [A T G A C G A A A A G C G A G C C T A]

It is possible to build a binary descriptor containing the position of all the mismatches

M [i] = [0 0 0 0 0 1 0 0 1 0 0 1 0 0 1 0 0 1 1]

Following Datta and Asif [24] we used the Positional Count Function (PCF) to count the number of 1's at phase s in the w-bit parsed words. Using a wordsize (w) of 3, C_3_^bname ^(2) is the count of 1 s (mismatches) in the binary descriptor name parsed in non overlapping 3-bin words (putative codons) at phase 2 (third position of putative codons).

For the alignment shown above the PCF functions calculated over all possible phases are



Using theorem 1 and 2 as indicated in (3) we express the spectral coding potential (SCP) of the alignment in term of Signal over Noise Ratio calculated as



Where the numerator represents the magnitude of DFT M [*K*] of the binary signal M [i] at frequency k = N/3 and the denominator is representing the noise expressed as the average value  of the squared magnitude |*Ã*[*k*]|^2^, (1 ≤ k ≤ N) of the DFT of M [i] excluding the dc component (A [*0*]).

3) StopCodons: We count the number of stop codons present in each potential reading frame of the longest gap-free block of the alignment (to avoid zero division errors we use a pseudocount starting from 1 and divide by the number of codons. We record values for the best frame (Stop-best) (the frame with the lowest number of stop codons) and the difference between the value for this frame and the mean value for the other 5 potential reading frames (Stop-delta).

4) Codon similarity score: For each potential reading frame in the longest gap-free block in the alignment, we calculate the mean of the codon similarity scores according to a codon substitution weight matrix derived from alignments of homologous vertebrate genes [25]. The statistic used (Codon-sim-ratio) is defined as the value for the highest scoring reading frame divided by the value for the lowest scoring reading frame.

5) Length of longest gap-free block in the alignment (GFB-length).

6) Nucleotide identity of longest gap-free block (GFB-ntID).

7) Percentage GC content of longest gap-free block in probe (GC-probe) and target (GC-target) sequence.

8) Number of transversions divided by the total number of substitutions in longest gap-free block (Tv/subs).

9) Percentage potential amino acid identity is calculated for each frame in the longest gap-free block and both the highest value (aaID-best) and the ratio of this value with the mean value of the other potential reading frames (aaID-ratio) are recorded.

### Support Vector Machine

We have used the SVM implemented in the software LIBSVM 2.82 [16] and have employed the Radial Basis Function (RBF) kernel in all analyses. For feature scaling we use the svm-scale program provided with the package. Optimization of the parameters C and G was performed using the grid-search method implemented in the python script grid.py provided with the software. In all cases, SVM training and prediction were performed with the command line option "-b 1" allowing the SVM to export probability estimates associated with classifications.

### Analyses of Features

Correlation matrices and the associated pictures, maximum frame score independence tests and feature relevance analyses were conducted using custom R language scripts. Statistical analyses of ROC curves were performed using the STaR software [18].

## Authors' contributions

MR conceived several features, extracted all data used, performed alignments, implemented all code relevant to feature extraction and analyses, and contributed to drafting of the manuscript. GP conceived the study and contributed to the preparation of the manuscript, DSH managed the study, provided scripts for automating the SVM and data post-processing and contributed to the preparation of the manuscript.

## Supplementary Material

Additional file 1 **Additional methods, figures and tables**. Methods for tests of independence of maximum-scoring reading frames and comparison of CSTminer with the current method, Figure S1, tables S1-S4. Click here for file
