# Supplementary material for: Accurate discrimination of conserved coding and non-coding regions through multiple indicators of evolutionary dynamics
Source: BMC Bioinformatics. 2009 Sep 8;10:282. doi: 10.1186/1471-2105-10-282 (PMC2758873; doi:10.1186/1471-2105-10-282)
Supplement: Additional file 1 — Additional methods, figures and tables. Methods for tests of independence of maximum-scoring reading frames and comparison of CSTminer with the current method, Figure S1, tables S1-S4. [file 1471-2105-10-282-S1.doc]

**Re et al. Additional File 1:**

**Tests of independence of maximum-scoring reading frames**

Several of the features used here rely on characteristics expected of coding and non-coding sequences as a result of evolutionary constraints implied by the genetic code. Several such measures are calculated for each possible reading frame of alignments and either the highest scoring frame or a measure of difference of the highest scoring frames and scores from other possible reading frames are used as indicators of coding potential (see Materials and Methods). Furthermore, some pairs of features share elements of the formulae used in their calculation. For example, the Coding Potential Score (CPS), incorporates the ratio between synonymous and non-synonymous substitution frequencies (Ns/Nns) and amino acid similarity scores (AAsim). It is therefore expected that such measures should consistently identify the same maximum scoring reading frames in CSTs analysed. We performed statistical independence tests of the frames in which a subset of features (amino acid identity (AAid), amino acid similarity (AAsim), ratio of synonymous to non synonymous substitution frequencies (Ns/Nns-best), frequency of stop codons (Stop-best) and CPS) produced the maximum scores for long and short CSTs. For each pair of descriptors we constructed a 6 by 6 contingency table containing the number of the observed maximum scoring frame co-occurrences for all the possible frames and tested the hypothesis:

H0: D(X|Y)=D(X)

using the Cochran-Mantel-Haenszel test to assess the independence of the maximum-scoring frame of feature X given the observed maximum-scoring frame of feature Y. Results are reported in Table S1.

It is interesting to note that while the dependence of maximum-scoring reading frames of some feature pairs is evident both for coding and non-coding CSTs (eg. CPS-best and Ns/Nns-best, CPS-best and AAsim-best, AAsim-best and AAid-best) – due to shared components of formulae, some pairs of descriptors are statistically independent (with respect to the observed max frame) for non-coding CSTs and show clear dependence only for coding CSTs (as in the case of the Stop-best and AAid-best, Stop-best and Ns/Nns-best, CPS-best and Stop-best feature pairs).

The SpectrAlign feature does not provide a frame specific measure but detects dishomogeneities of substitution frequencies occurring between all frames. Other observed frame dependency patterns are independent of the length classes employed.

**Comparisons of CSTminer with the current method**:

In order to compare the performances of CSTminer and the method proposed here, we computed the areas under the curve using the predicted CP of all the CSTs belonging to the evaluation set produced by CSTminer and the new method. This test has been performed comparing the performances of the two methods in the prediction of long CSTs, short CSTs and all the evaluation set CSTs disregarding their length.  The statistical significance of the differences observed between the AUC scores were then assessed by using the non parametric Mann-Whitney test at 0.01 significance cutoff.

test set comparison:

CSTminer AUC:    current AUC:    Mann-Withney test

-----------------------------------------------------------------------

Long CSTs    0.933892    0.99269        2.65240e-22

Short CSTs    0.810800    0.95458        6.30707e-29

ALL      0.871634    0.980134    2.08694e-61

**Figure S1:**

**ROC curve comparisons for CSTminer and the current method using the test dataset:** Solid lines show the performance of CSTminer and dashed lines the performance of the new method.

**
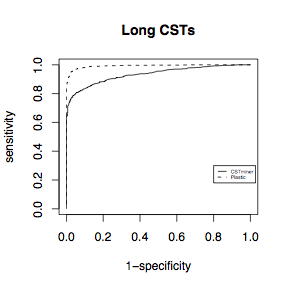

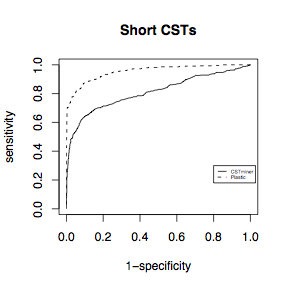
**

**
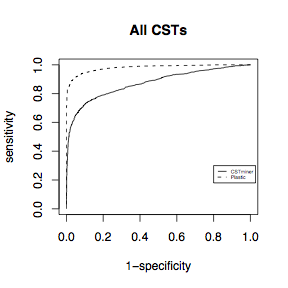
**

**Supplementary Tables**

**Table S1:** Cochran-Mantel-Haenszel tests of independence of the maximum-scoring frame of feature X given the observed maximum-scoring frame of feature Y. Chi squared scores and associated Pvalues for independence of maximum scoring frame of any feature, given the observed maximum scoring frame of another feature are reported.

**Table S2:** Area Under Curve (AUC) statistics for trained SVM and individual features using validation sets.

**Table S3**: Significance of SVM performance with respect to that of individual features for short and long validation tests using a non-parametric test of ROC curve characteristics.

**Table S4**: Significance of difference of performance between pairs of individual features for short and long validation tests using a non-parametric test of ROC curve characteristics.
